# The Quest for Trustworthy, Probative, and Creative Research in Geroscience

**DOI:** 10.1093/geroni/igaf122.1106

**Published:** 2025-12-31

**Authors:** David Allison

**Affiliations:** Baylor College of Medicine, Houston, Texas, United States

## Abstract

In the field overall, in the enjoinders of luminaries and funders such as the Hevolution Foundation, AFAR, the Academy of Healthspan and Longevity Research, the National Academy of Medicine, and the strategic plans and executive orders from the NIH and federal government, there is a consistent theme of the need to increase rigor and trustworthiness in biomedical research in general and in geroscience. We are called upon to increase rapidity of translation and the probative nature of research being conducted in geroscience. In this presentation, I will discuss ways we can make geroscience ever more probative, ever more impactful, build on the tremendous momentum that exists now to promote creative ground-breaking research, and do it all while increasing the rigor and trustworthiness of our science. I will focus both on research training in this area, as well as research funding and execution.

